# Insights into the ecological generalist lifestyle of *Clonostachys* fungi through analysis of their predicted secretomes

**DOI:** 10.3389/fmicb.2023.1112673

**Published:** 2023-02-16

**Authors:** Edoardo Piombo, Micol Guaschino, Dan Funck Jensen, Magnus Karlsson, Mukesh Dubey

**Affiliations:** ^1^Department of Forest Mycology and Plant Pathology, Swedish University of Agricultural Sciences, Uppsala, Sweden; ^2^Department of Agricultural, Forest and Food Sciences (DISAFA), University of Torino, Grugliasco, Italy

**Keywords:** biocontrol, *Clonostachys*, mycoparasitism, small secreted cysteine-rich proteins, effector, CFEM proteins, antagonism, secretome

## Abstract

**Introduction:**

The fungal secretome comprise diverse proteins that are involved in various aspects of fungal lifestyles, including adaptation to ecological niches and environmental interactions. The aim of this study was to investigate the composition and activity of fungal secretomes in mycoparasitic and beneficial fungal-plant interactions.

**Methods:**

We used six *Clonostachys* spp. that exhibit saprotrophic, mycotrophic and plant endophytic lifestyles. Genome-wide analyses was performed to investigate the composition, diversity, evolution and gene expression of *Clonostachys* secretomes in relation to their potential role in mycoparasitic and endophytic lifestyles.

**Results and discussion:**

Our analyses showed that the predicted secretomes of the analyzed species comprised between 7 and 8% of the respective proteomes. Mining of transcriptome data collected during previous studies showed that 18% of the genes encoding predicted secreted proteins were upregulated during the interactions with the mycohosts *Fusarium graminearum and Helminthosporium solani*. Functional annotation of the predicted secretomes revealed that the most represented protease family was subclass S8A (11–14% of the total), which include members that are shown to be involved in the response to nematodes and mycohosts. Conversely, the most numerous lipases and carbohydrate-active enzyme (CAZyme) groups appeared to be potentially involved in eliciting defense responses in the plants. For example, analysis of gene family evolution identified nine CAZyme orthogroups evolving for gene gains (*p* ≤ 0.05), predicted to be involved in hemicellulose degradation, potentially producing plant defense-inducing oligomers. Moreover, 8–10% of the secretomes was composed of cysteine-enriched proteins, including hydrophobins, important for root colonization. Effectors were more numerous, comprising 35–37% of the secretomes, where certain members belonged to seven orthogroups evolving for gene gains and were induced during the *C. rosea* response to *F. graminearum or H. solani*. Furthermore, the considered *Clonostachys* spp. possessed high numbers of proteins containing Common in Fungal Extracellular Membranes (CFEM) modules, known for their role in fungal virulence. Overall, this study improves our understanding of Clonostachys spp. adaptation to diverse ecological niches and establishes a basis for future investigation aiming at sustainable biocontrol of plant diseases.

## 1. Introduction

Fungal secreted proteins (secretome) play an important role in fungal biology and adaption to various ecological niches, and environmental interactions ranging from mutualism to parasitism and interference competition (Druzhinina et al., 2012; Pellegrin et al., 2015; Lu and Edwards, 2016). Genes encoding secreted proteins typically encompass 4–15% of the total gene numbers in fungal genomes (Girard et al., 2013; Pellegrin et al., 2015). These proteins are classified into various functional groups such as carbohydrate-active enzymes (CAZymes), proteases, lipases and oxidoreductases needed for nutrient acquisition, self-protection and biotic interactions with microbes, plants and animals (Kim et al., 2016; Guzmán-Guzmán et al., 2017; Feldman et al., 2020). Moreover, 40–60% of predicted fungal secretome proteins are typically shorter than 300 amino acids (aa) and are referred to as small secreted proteins (SSPs; Pellegrin et al., 2015; Kim et al., 2016). SSPs are often cysteine-rich, lack known protein modules or catalytic domains and certain members act as effectors mediating communication between organisms ranging from beneficial to detrimental interactions (Kim et al., 2016; Selin et al., 2016).

SSPs are mainly studied in the context of pathogenic fungal-plant interactions, and their composition and role in parasitic fungal-fungal and beneficial fungal-plant interactions is still poorly investigated. In fungi used for biological control of plant diseases, for example members of the *Trichoderma* and *Clonostachys* genera, the function of certain SSPs including hydrophobins, cerato-platanins and LysM module-containing proteins in regulating interactions with fungal hosts (mycohosts) and plant hosts are shown (Dubey et al., 2012, 2014; Guzmán-Guzmán et al., 2017; Ramírez-Valdespino et al., 2019; Jensen et al., 2021). In addition, a SSP family with Common in Fungal Extracellular Membranes (CFEM) modules has been identified in *T. atroviride* (Kulkarni et al., 2003; Druzhinina et al., 2012; Zhang et al., 2015; Guzmán-Guzmán et al., 2017). Certain members of this family have been shown to be induced during interactions with host plants indicating their roles as putative effector proteins (Guzmán-Guzmán et al., 2017). A role of CFEM-containing proteins in fungal pathogenesis has been demonstrated in the plant pathogenic fungi *Botrytis cinerea*, *Magnaporthe oryzae*, and *Colletotrichum graminicola* (Kou et al., 2017; Zhu et al., 2017; Gong et al., 2020).

*Clonostachys* spp. including *C. rosea*, *C. byssicola*, *C. chloroleuca*, *C. rhizophaga* and *C. solani*, are filamentous ascomycetes fungi with a multi-trophic mode of lifestyle. These fungi can be found as saprotrophs in various ecological niches including soil and dead organic matter (Moreira et al., 2016; Jensen et al., 2021). Certain species can thrive in rhizospheres where they can colonize the plant root surfaces and establish a beneficial relationship with the plant host as endophytes (Sutton et al., 2002; Karlsson et al., 2015; Saraiva et al., 2015; Maillard et al., 2020; Jensen et al., 2021). In addition, these species can live as necrotrophic mycoparasites by killing and feeding on their mycohosts (Alvindia and Natsuaki, 2008; Dugan et al., 2012; Sun et al., 2018; Jensen et al., 2021). The antagonistic ability of certain strains of *C. rosea* against plant parasitic nematodes has also been reported (Iqbal et al., 2018a,b, 2020). To succeed in these distinct ecological niches, *Clonostachys* spp. have evolved capabilities for decomposition of organic materials, competition with other microorganisms for nutrients and space in soil and rhizosphere, and interference competition through antibiosis and mycoparasitism (Morandi et al., 2000; Li et al., 2002; Saraiva et al., 2015; Sun et al., 2017; Fatema et al., 2018). Due to these properties, certain *Clonostachys* strains are used as efficient biological control agents against fungal plant diseases in agricultural and horticultural production systems (Jensen et al., 2000; Sutton et al., 2002; Xue et al., 2008; Cota et al., 2009; Salamone et al., 2018).

The ability of *Clonostachys* spp. to inhabit broad ecological niches is reflected by their genomic characteristics (Karlsson et al., 2015; Broberg et al., 2021). For example, copy number of genes coding for enzymes associated with biosynthesis of specialized metabolites such as polyketide synthases, non-ribosomal peptide synthetase and cytochrome P450 monooxygenases are expanded in *C. rosea* compared to plant pathogenic *Fusarium* spp. and mycoparasitic *Trichoderma* spp. (Karlsson et al., 2015; Broberg et al., 2021). Similarly, the ATP-binding cassette (ABC) and major facilitator superfamily (MFS) membrane transporter gene families, associated with efflux of endogenous and exogenous specialized metabolites, are also expanded (Karlsson et al., 2015; Nygren et al., 2018; Broberg et al., 2021). Among the CAZyme gene families, auxiliary activity (AA) family 9 lytic polysaccharide monooxygenases, AA7 gluco-and chitooligosaccharide oxidases, AA3 glucose-methanol-choline oxidoreductases, polysaccharide lyase family 1 (PL1) pectin/pectate lyases and certain proteases are also evolving under selection for increased gene copy numbers (Karlsson et al., 2015; Atanasova et al., 2018; Iqbal et al., 2018a,b; Broberg et al., 2021). However, analysis of gene family evolution of genes specifically coding for secreted proteins in *Clonostachys* is yet to be comprehensively investigated.

In this study, we performed prediction and in-depth analysis of the secretomes of six *Clonostachys* spp., including *C. byssicola*, *C. chloroleuca*, *C. rhizophaga*, *C. rosea*, *C. solani* and *Clonostachys* sp. CBS 192.96 with the hypothesis that the composition of *Clonostachys* spp. secreteome is shaped to accomplish their saprotrophic, mycotrophic and plant endophytic lifestyles. Our analysis was focused on predicted lipases, proteases, oxidoreductases, CAZymes, cysteine-rich SSPs, putative effectors and CFEM proteins and their possible roles in mycoparasitic and beneficial fungal-plant interactions. This revealed the presence of many proteins with a known role in antagonism against mycohosts and nematodes, including chitinases, endopolygalacturonases, subtilisin-like peptidases and phospholipases A. Moreover, several enzyme classes including hemicellulose and cellulose degradation enzymes, SSPs and effectors with putative role in fungus-plant interactions were identified.

## 2. Materials and methods

### 2.1. Prediction of fungal secretomes

Based on the genome sequences of *C. rosea* IK726 (Genbank: GCA_902827195.2), *C. byssicola* CBS 245.78 (GCA_902006505.2), *C. chloroleuca* CBS 570.77 (GCA_902074915.2), *C. rhizophaga* CBS 906.72A (GCA_902077795.2), *C. solani* 1703 (GCA_902141235.2) and *Clonostachys* sp. CBS 192.96 (Karlsson et al., 2015; Broberg et al., 2018, 2021; Supplementary file 1), the predicted secretomes were generated using procedures described previously (Gogleva et al., 2018). In short, SignalP ver. 4.0 (Petersen et al., 2011) was used to predict secretion signal peptides, while proteins with transmembrane domains were identified with TMHMM ver. 2 (Krogh et al., 2001). TargetP ver. 2 (Armenteros et al., 2019) was used to identify proteins putatively targeted to mitochondria, while PredGPI (Pierleoni et al., 2008) was used to predict proteins with a glycosylphosphatidylinositol (GPI) anchor. The complete bash script is available as Supplementary file 2. Proteins with predicted signal peptides but lacking transmembrane domains, GPI anchors or mitochondrial targeting signatures, were considered to be secreted. OrthoFinder ver. 2.5.2 (Emms and Kelly, 2019) was used to identify orthogroups of the secreted proteins. For comparative purposes, secretome prediction and InterProScan annotation was performed on the genomes of *Neurospora crassa* OR74A (GCA_000182925.2), *T. atroviride* IMI 206040 (GCA_000171015.2), *T. reesei* QM6a (GCA_000167675.2 v2.0), *T. virens* Gv29-8 (GCA_000170995.2), *F. graminearum* PH-1 (GCA_000240135.3), *F. verticillioides* 7600 (GCA_000149555.1) and *F. vanetteni* 77–13-4 (Coleman et al., 2009).

### 2.2. Functional annotation and gene ontology enrichment analyses

Gene ontology (GO) enrichment analyses were performed using the agriGO toolkit (Tian et al., 2017) with the Fisher statistical test and maximum adjusted *p*-value of 0.05. The FDR adjustment method was used to adjust the *p*-value. The GO annotation for the proteome of *C. rosea* was obtained from a previous study (Piombo et al., 2021).

The composition of the *Clonostachys* secretomes was analyzed with focus on predicted proteases, lipases, CAZymes, oxidoreductases, cysteine rich proteins and effectors, due to their potential role in environmental interactions. InterProScan v. 5.46–81 (Jones et al., 2014) was used to predict modules in the proteomes, and lipases, oxidoreductases and proteases were specifically identified based on the presence of InterProScan modules. Lipases and oxidoreductases were further classified in classes depending on the type of detected module, while proteases were classified according to the Merops database (Rawlings et al., 2010), using the BLAST algorithm (Altschul et al., 1990). EffectorP 3.0 was used to predict putative effectors (Sperschneider and Dodds, 2021), and the dbCAN meta server was used to predict CAZymes using both the HMMER and DIAMOND tools (Buchfink et al., 2015; Zhang et al., 2018).

### 2.3. CFEM identification and analysis

Proteins predicted to contain CFEM modules using InterProScan v. 5.46–81 (Jones et al., 2014) were considered to be CFEM proteins. For phylogenetic analysis, CFEM proteins were aligned with mafft v.7.453 in E-INS-I mode, suggested for sequences containing large unalignable regions (Katoh and Standley, 2013), and the phylogenetic trees were generated with iqtree v.2.1.3 (Nguyen et al., 2015) with 500 bootstrap replicates and the option “MFP” (ModelFinder) to find the best substitution model. Visualization was carried out with Figtree v.1.4.4 (Rambaut, 2018). The same programs were used for the phylogenetic analysis of the concatenated CFEM modules of each species, but mafft was used in L-INS-I mode, recommended when working with less than 200 sequences.

### 2.4. Study of gene family evolution

Computational analysis of gene family evolution (CAFE) v.5 (Mendes et al., 2020) was used to estimate accelerated rates of gene gain or loss, associated with lineages. The significance threshold was set at 0.05. The phylogenetic tree necessary for CAFE analysis was obtained as described in Broberg et al. (2021), using concatenated gene sequences of ATP citrate lyase (*acl1*), RNA polymerase II large subunit (*rpb1*), translation elongation factor 1-α (*tef1*) and β-tubulin (*tub*) for the considered species.

### 2.5. Mining of gene expression data

To investigate transcriptional regulation of genes coding for secreted proteins, differentially expressed genes of *C. rosea* interacting with the mycohosts *Botrytis cinerea*, *F. graminearum* and *Helminthoisporum solani* were retrieved from four previously published studies (Lysøe et al., 2017; Demissie et al., 2018, 2020; Nygren et al., 2018). When the studies used different version of the *C. rosea* genome, proteins from different versions were considered the same if they had a match in a BLAST analysis with 90% minimum identity and query coverage. Each proteome was used as both query and database for the BLAST analyses, and only proteins with a match in both database-query combinations were accepted.

## 3. Results

### 3.1. Prediction of *Clonostachys* secretomes and mining of gene expression data

**Figure 1:**
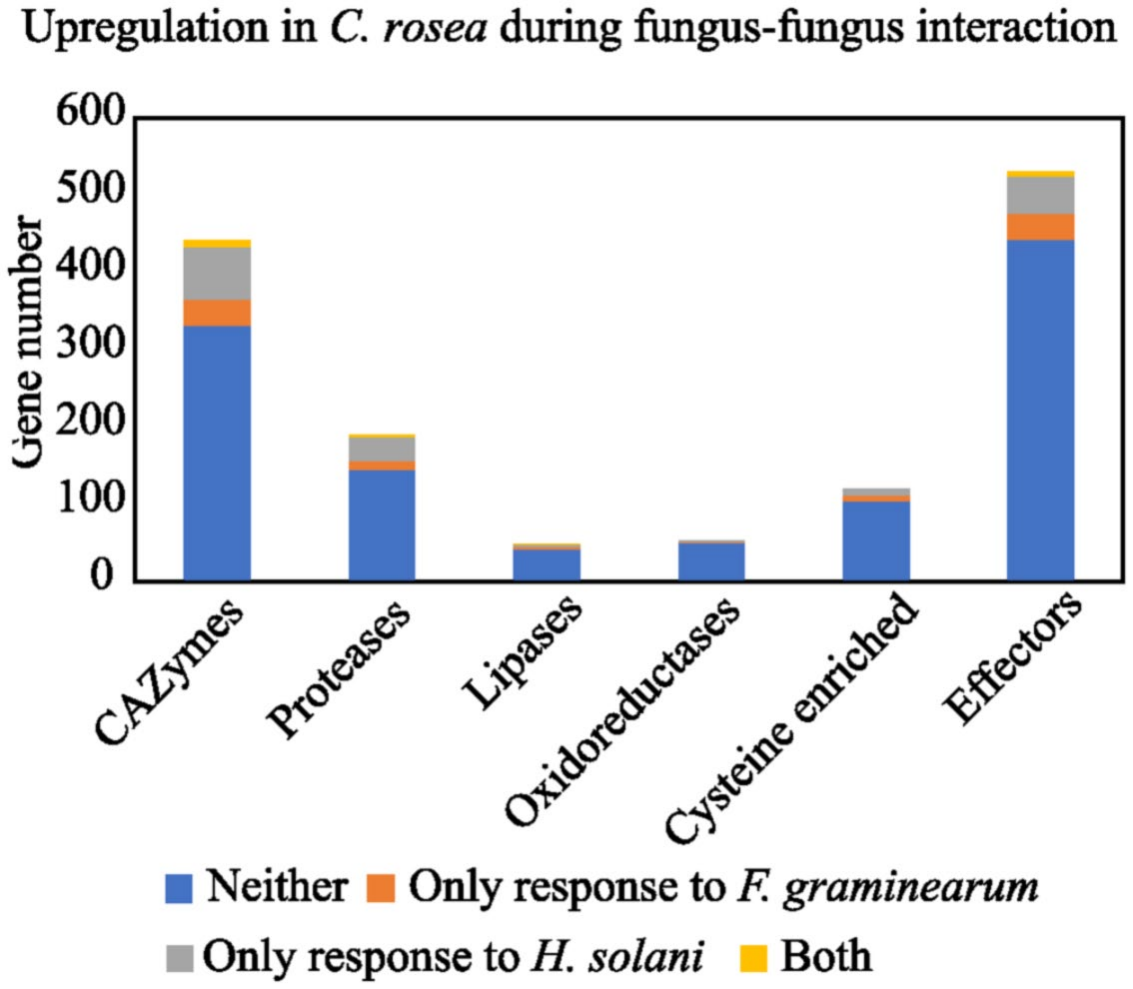
Number of *C. rosea* transcripts, coding for secreted proteins, upregulated during contact with *H. solani* and/or *F. graminearum*. The numbers are shown for CAZymes, proteases, lipases, oxidoreductases, cysteine enriched proteins and effectors. The gene expression data was retrieved from previously performed transcriptome analysis of *C. rosea* during interactions with *H. solani* or *F.* graminearum (Lysøe et al., 2017; Demissie et al., 2018, 2020; Nygren et al., 2018).

**Figure 2:**
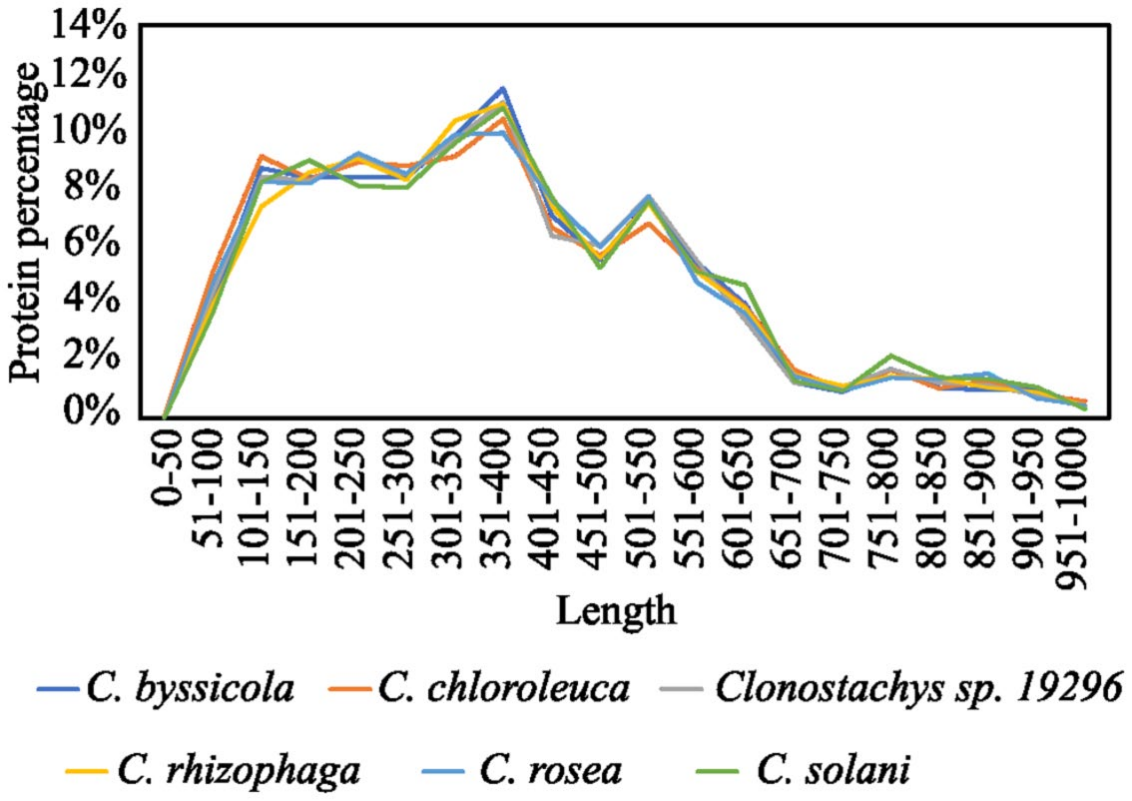
Size distribution of *Clonostachys* predicted secretomes.

The secretomes of six *Clonostachys* spp. was predicted to contain 1,428 to 1,498 proteins, amounting to between 7.1% (*C*. *rosea*) and 8.0% (*C*. *byssicola*) of their proteomes (Table 1; Supplementary file 3). In the considered *Trichoderma* spp., the proportion of secreted proteins in their predicted secretomes accounted for 4.9% in *T. reesei* to 5.6% in *T. atroviride*. More than 85% of the *Clonostachys* spp. secreted proteins were less than 600 aa in length (Figure 1), among which a majority of proteins (56%) were 100–400 aa in length with the highest proportion (around 12%) at 350–400 aa. No differences were found in proportion and length distribution of secreted proteins between the analyzed *Clonostachys* species. Mining of available RNA-seq data (Lysøe et al., 2017; Demissie et al., 2018, 2020; Nygren et al., 2018) identified 274 genes upregulated in *C. rosea* during interaction with the mycohosts *F. graminearum* or *H. solani* (Figure 2; Supplementary Table S1).

**Table 1:** Summary of predicted secretomes in the *Clonostachys* spp.

	*C. bys*	*C. chl*	*Clono* sp.	*C. rhi*	*C. ros*	*C. sol*	*T. atr*	*T. ree*	*T. vir*
Proteome	18,541	19,658	18,459	18,962	21,246	18,093	11,816	9,111	12,406
GPI anchors	178	164	180	173	175	178	80	72	93
Secretome	1,475 (7.96%)	1,495 (7.61%)	1,428 (7.74%)	1,467 (7.74%)	1,498 (7.05%)	1,435 (7.93%)	664 (5.62%)	449 (4.93%)	667 (5.38%)
Proteases	196	187	192	195	191	193	54	42	54
Lipases	56	57	58	60	49	54	11	7	12
CAZymes	445	459	434	453	444	428	228	153	214
Cysteine-enriched secreted proteins	126	148	124	120	121	118	99	52	88
Oxidoreducteses	62	55	53	54	53	54	6	6	10
Effectors	517	560	510	517	530	500	262	154	260
Others	602	607	578	592	656	599	178	128	190

Species abbreviations: *C. bys*, *Clonostachys byssicola*; *C. chl*, *Clonostachys chloroleuca*; *Clono* sp., *Clonostachys* sp. *CBS 192.96*; *C. rhi*, *Clonostachys rhizophaga*; *C. ros*, *Clonostachys rosea*; *C. sol*, *Clonostachys solani*; *T. atr*, *Trichoderma atroviride*; *T. ree*, *Trichoderma reesei*; *T. vir*, *Trichoderma virens*.

### 3.2. Gene ontology enrichment analysis of predicted *Clonostachys* secretomes

**Figure 3:**
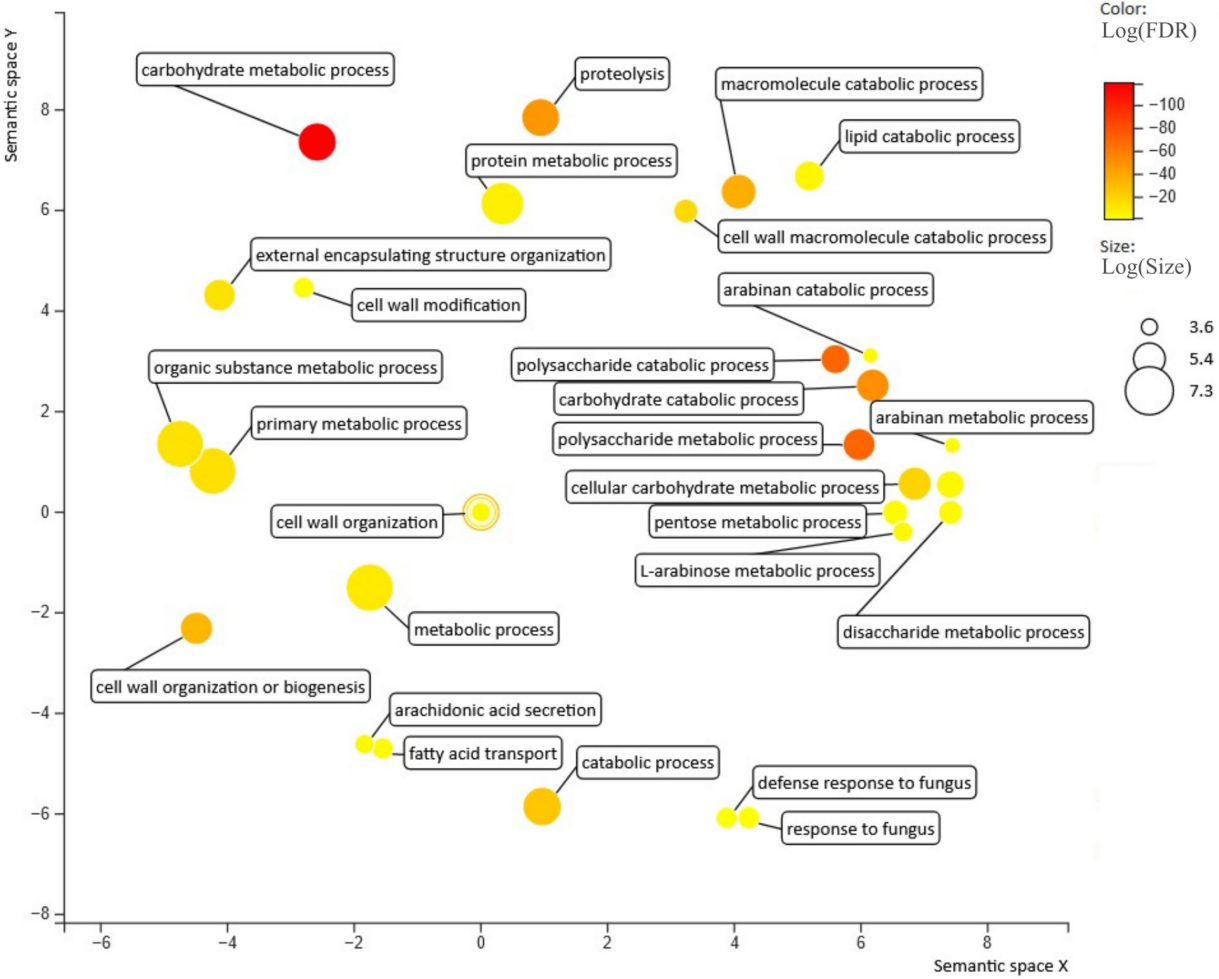
Biological processes enriched in the secretome predicted for *C. rosea*. Figure generated with revigo (Supek et al., 2011).

GO enrichment analysis based on the annotated *C. rosea* proteome (Piombo et al., 2021) revealed that 57 biological processes were enriched (*p* ≤ 0.05) in the *C. rosea* secretome in respect to the rest of the proteome (Figure 3). The analysis was run on the proteome of *C. rosea* as it had the best available functional annotation among the considered *Clonostachys* spp., and had underwent multiple functional analyses in previous studies (Broberg et al., 2018, 2021; Piombo et al., 2021). The majority of the enriched biological processes were related to metabolic and catabolic activity on several compounds including carbohydrates, proteins and lipids. The terms response to fungus (GO: 0009620), defense response to fungus (GO: 0050832), cell wall organization (GO: 0071555), cell wall organization or biogenesis (GO: 0071554) were also enriched (Figure 3).

### 3.3. Composition of predicted *Clonostachys* secretomes

#### 3.3.1. Carbohydrate-active enzymes

A large number of CAZymes (428–459 genes) comprising 30% of the secretomes on average, were detected in the *Clonostachys* species. The percentage of CAZymes in *Clonostachys* spp. was lower compared to the predicted proportion of 30 to 35% in the considered *Trichoderma* spp. (Table 1). The highest number of predicted CAZymes in *Clonostachys* spp. were classified as GH43 (arabinofuranosidases, arabinases and xylosidases), followed by AA7 (glucooligosaccharide oxidases), AA9 (lytic polysaccharide monooxygenases), AA3 (glucose-methanol-choline oxidoreductases), GH5 (hydrolases with many substrates) and GH28 (polygalacturonases) (Supplementary Table S2). The number of detected GH18 chitinases was between six and nine, while three or four GH20 N-acetylglucosaminidases were identified (Supplementary Table S2).

CAFE analysis identified ten CAZyme families evolving for gene gains or losses (*p* ≤ 0.05) in the *Clonostachys* spp. (Table 2). These included different classes of enzymes degrading cellulose, glucan, xylan, trehalose and other components of the fungal and plant cell wall, including AA9, GH3, GH15, GH16, GH31 and GH78 (*p* ≤ 0.037). The highest number of secreted CAZyme families (five families) evolving for gene gains were identified in *C. chloroleuca* followed by three families in *C*. *rosea* (Table 2). Intriguingly, only GH15 was evolving for gene gains or losses in *Clonostachys* sp. CBS 192.96, with a significant (*p* = 0.001) decrease from five genes in the ancestral species to one gene in *Clonostachys.* sp. CBS 192.96. Conversely, only family AA9 was evolving for gene gains or losses in *C. solani*, with a decrease from 24 to 16 genes compared with the ancestral lineage (*p* < 0.001). Mining of gene expression data also revealed that family AA9 was the most represented class by far among the CAZymes significantly upregulated in *C. rosea* during the interaction with *F. graminearum* or *H. solani* (Supplementary Table S1), with 12 out of 111 upregulated CAZymes belonging to this class, followed by GH7, GH12 and GH28 with 5 members each.

**Table 2:** Gene numbers in gene families evolving for gene gains in *Clonostachys* spp.

Gene family^a^	Description	*C. bys*	*C. chl*	*Clono* sp.	*C. rhi*	*C. ros*	*C. sol*	Ancestor sp.
AA7	Glucooligosaccharide oxidase	31	23	26	27	26	29	27
AA9	Lytic polysaccharide monooxygenase	27	32	25	25	27	16	24
AA1	Multicopper oxidases	4	5	2	4	3	2	3
CE1	Esterase (variable substrates)	7	11	9	8	8	7	8
GH15	Glucoamylase, trehalase	8	7	1	7	6	8	5
GH16	Glucanase, xylanase	9	14	9	11	10	8	9
GH3	Glucanase, xylanase	14	8	12	11	14	15	13
GH31	Glucosidase, galactosidase	3	3	4	4	7	4	4
GH39	Variable substrate	3	2	4	3	5	5	4
GH78	α-L-rhamnosidase	4	6	6	4	3	5	5
Lipase 3	Lipase	5	5	5	8	3	5	5
GDSL Esteraselipase EXL3	Lipase	10	14	6	6	6	6	7
S10	Serine carboxypeptidase	7	4	4	4	5	5	4
M43B	Carboxypeptidase	9	6	7	9	7	9	8
S33	Serine protease	9	9	8	11	8	6	8

*C. bys, Clonostachys byssicola*; *C. chl*, *Clonostachys chloroleuca*; *Clono* sp., *Clonostachys* sp. *CBS192.96*; *C. rhi*, *Clonostachys rhizophaga*; *C. ros*, *Clonostachys rosea*; *C. sol*, *Clonostachys solani*.

Gene numbers boxed in black indicates a significant (*p* ≤ 0.05) expansion, while gene numbers boxed in grey indicates a significant (*p* ≤ 0.05) contraction of gene family size compared with the most recent ancestor (Broberg et al., 2021).

A cut-off value of 5 genes was used to determine the gene family evolving for gene gains.

a Carbohydrate-active enzyme gene family classification is based on dbCAN meta server, protease gene family classification is based on merops database. Lipase gene family classification is based on InterProScan analysis.

#### 3.3.2. Proteases, lipases, and oxidoreductases

On average 12% of the *Clonostachys* secretomes (187–196 genes) were classified as proteases of several Merops classifications (Rawlings et al., 2012; Table 1), while only around 8.5% of the secretome (41–54 genes) was comprised of proteases in *Trichoderma* spp. (Table 1). The dominant groups in *Clonostachys* spp. were serine proteases (S8A serine endopeptidase subtilisins, S33 serine proteasesand S1A chymotrypsins), carboxypeptidases (M14A) and metallo-endopeptidases (M43B), all families with many more members in *Clonostachys* spp. than in *Trichoderma* spp. (Supplementary Table S2). Gene family evolution analysis identified three protease families (S10, S33 and M43B) as evolving for gene gains or losses in the considered *Clonostachys* spp. (Table 2). The S10 serine carboxypeptidase gene family was significantly (*p* = 0.004) expanded from four to seven genes in *C*. *byssicola*, while subfamily M43B was significantly contracted in *C. chloroleuca* (*p* = 0.004) compared to the number in the ancestral species, while family S33 was expanded in *C. rhizophaga* and contracted in *C. solani* (Table 2). Among the 191 secreted proteases identified in *C. rosea*, 46 were upregulated during the interactions with *F. graminearum* or *H. solani* (Figure 2; Supplementary Table S1). The most frequent classes in this subgroup were serine endopeptidases of family S8A and S1A, with six genes each.

Predicted lipases amounted to 3.8% of the total secretomes on average in *Clonostachys* spp., but only 1.7% in *Trichoderma* spp. (Table 1). The most represented groups in *Clonostachys* spp. were phospholipases A2, lipases 5, lysophospholipases L1 and the GDSL-like Lipase/Acylhydrolase family (Supplementary Table S2). Lipases 3 and GDSL esteraselipase exl3 were predicted to be evolving for gene gains. The gene copy number of the lipases 3 gene family was expanded in *C. rhizophaga* (*p* = 0.001) and contracted (*p* = 0.013) in *C. rosea*, while the gene copy number of the GDSL esteraselipase exl3 gene family was expanded (*p* = 0.001) in *C. chloroleuca* but contracted (*p* ≤ 0.031) in *C. rhizophaga* and *C. rosea* (Table 2). Seven lipase encoding genes were significantly upregulated in *C. rosea* during the interactions with *F. graminearum* or *H. solani* (Figure 1; Supplementary Table S1).

Numerous predicted oxidoreductases were detected in the *Clonostachys* secretomes, ranging from 62 in *C. byssicola* to 53 in *C. rosea* and *Clonostachys* sp. CBS 192.96. This amounted to 3.8% of the secretome on average, against the 1.3% in *Trichoderma* spp. (Table 1). The most represented class was the AA3 glucose-methanol-choline oxidoreductases, which amounted to around one third of the total in all the considered *Clonostachys* species. The second most frequent family in *Clonostachys* spp. was the flavin-containing amine oxidoreductases, which varied from 10 genes in *C. chloroleuca* to six genes in *C. rosea* (Supplementary Table S2). Three oxidoreductase encoding genes were upregulated in *C. rosea* during the interactions with *F. graminearum* or *H. solani* (Figure 1; Supplementary Table S1).

#### 3.3.3. Cysteine-enriched proteins and effectors

On average, 8.5% of the secretomes (118 out of 1478 proteins) consisted of proteins shorter than 300 aa and with more than 4% of cysteine residues and were considered cysteine-enriched. Conversely, at least 11.5% of secreted proteins were classified as cysteine-enriched in all *Trichoderma* spp. (Table 1). The GO enrichment analysis of cysteine-enriched proteins showed enrichment (*p* ≤ 0.05) in molecular functions related to lytic activity, specifically carbon–oxygen lyase activity (GO:0016835), carbon–oxygen lyase activity acting on polysaccharides (GO:0016837), pectate lyase activity (GO:0030570) and lyase activity (GO:0016829).

**Figure 4:**
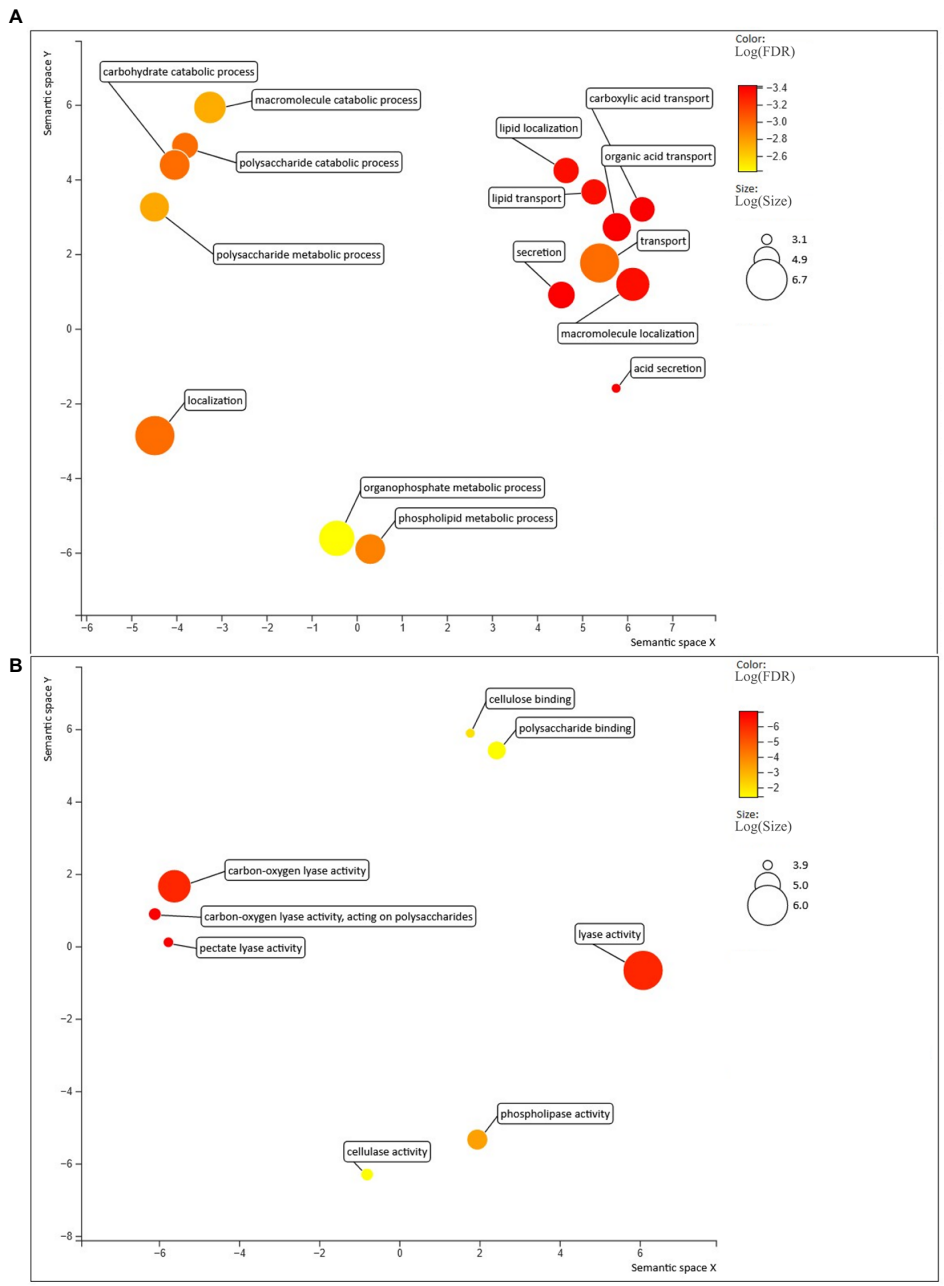
Biological processes **(A)** and molecular functions **(B)** enriched in the predicted effectors, compared with the rest of the *C. rosea* secretome. Figure generated with Revigo (Supek et al., 2011).

More than one third of the secretomes (500 of 563 proteins, 36% on average) consisted of putative effectors, while this amount was on average 40% in *Trichoderma* spp. (Table 1). However, less than half of the detected effectors contained a known InterProScan amino acidic motif, while the rest was uncharacterized. Several known effector classes already identified in *Trichoderma* spp. (Guzmán-Guzmán et al., 2017) such as serine proteases, metalloproteases, LysM proteins, cerato-platanins, thioredoxins and CFEM proteins, were detected among the predicted effectors in *Clonostachys* spp. (Supplementary Table S2). Gene expression analysis identified 88 *C. rosea* effector genes to be induced in response to *F. graminearum* or *H. solani* (Figure 2; Supplementary Table S1). Predicted effector proteins were enriched (*p* ≤ 0.05) in GO terms related to cell wall degradation and penetration in plant tissues, including cellulase activity (GO:0008810), pectate lyase activity (GO:0030570) and polysaccharide catabolic process (GO:0000272), but also in GO terms referring to transport and localization of lipids and acids, such as acid secretion (GO:0046717) and lipid transport (GO:0006869) (Figure 4).

Between 93% (*Clonostachys* sp. CBS 192.96) and 98% (*C. rhizophaga*) of the cysteine-enriched secreted proteins were also predicted to be effectors (Supplementary Figure S1). One particular class of cysteine-enriched proteins was hydrophobins, where *C. chloroleuca* had the highest number of secreted proteins (11 proteins) among the analyzed species, followed by *C. rosea* with six proteins and *C. solani* with three predicted secreted hydrophobins (Supplementary Table S2).

### 3.4. Analysis of evolution of gene family composition

An orthofinder analysis grouped the genes encoding predicted secreted proteins of the *Clonostachys* spp. into 1,547 orthogroups, of which 816 contained at least one gene from each included species (Supplementary Table S3). CAFE analysis identified a total of 38 orthogroups (involving 74 genes) evolving for gene gains (*p* ≤ 0.05) (Table 3). Twenty-one orthogroups were found to consist of 37 *C. rosea genes* coding for various families of glycosyl hydrolases. Among those, eight predicted proteins contained additional carbohydrate-binding modules (CBMs) and 13 were found to be differentially expressed during interspecific interaction with *F. gramineaum* or *H. solani* (Table 3). Seven orthogroups containing 15 *C. rosea* genes were predicted to encode various families of proteases including cuticle-degrading proteases, serine-type endopeptidases, subtilisin-like proteases and metallocarboxypeptidases. Orthogroup OG0000107 contained aspartyl proteases, a class involved in the response to both fungi and plants in *Trichoderma* spp. (Viterbo et al., 2004; Kredics et al., 2005; Table 3). Furthermore, nine orthogroups composed of 16 putative *C. rosea* effectors were identified, of which seven were found to be induced in response to *F. graminearum* or *H. solani* (Table 3).

**Table 3:** Orthogroups evolving for gene gains predicted by Orthofinder v. 2.5.2 in *Clonostachys* secretomes.

Orthogroup^a^	Value of *p*	Transcript	Annotation	Predicted function	Expression^b^
OG0000001	0.027	CRV2T00017963_1		Cuticle-degrading protease	
	0.027	CRV2T00016760_1		Serine-type endopeptidase	
	0.027	CRV2T00016723_1		Subtilisin-like protease	
OG0000005	0.018	CRV2T00004568_1	GH28	Hydrolase	
	0.018	CRV2T00002418_1	GH28	Polygalacturonase	
OG0000014	0	CRV2T00003403_1	Predicted effector	Cellulose binding protein	*F. graminearum*
	0	CRV2T00020679_1	Predicted effector	Cellulose binding protein	
	0	CRV2T00022039_1	Predicted effector	Cellulose binding protein	
	0	CRV2T00022042_1	Predicted effector	Cellulose binding protein	
	0	CRV2T00013751_1		Uncharacterized	
OG0000016	0.045	CRV2T00013015_1	GH54 + CBM13 + CBM42	Arabinofuranosidase	*F. graminearum, H. solani*
	0.045	CRV2T00015338_1	GH54 + CBM13 + CBM42 Predicted effector	Arabinofuranosidase	*F. graminearum*
OG0000018	0.009	CRV2T00007061_1		Uncharacterized	
	0.009	CRV2T00013113_1		Uncharacterized	
	0.009	CRV2T00017144_1		Uncharacterized	
OG0000022	0.001	CRV2T00010462_1	GH2	Beta-galactosidase	*F. graminearum, H. solani*
OG0000023	0.001	CRV2T00008810_1		Serine-type carboxypeptidase	*H. solani*
OG0000024	0.004	CRV2T00000095_1	GH12 Predicted effector	Cellulolytic endoglucanase	*F. graminearum*
OG0000029	0	CRV2T00011057_1	CE5 + CBM1 Predicted effector	Esterase precursor protein	*F. graminearum*
OG0000057	0.017	CRV2T00004531_1	GH43_14	Glicoside hydrolase	
	0.017	CRV2T00004861_1	GH43_14	Glicoside hydrolase	
OG0000058	0.004	CRV2T00001563_1	Predicted effector	Phospholipase A2	*H. solani*
	0.004	CRV2T00008733_1	Predicted effector	Phospholipase A2	
OG0000064	0	CRV2T00008520_1	GH31	Glicoside hydrolase	*H. solani*
	0	CRV2T00009927_1	GH31	Glicoside hydrolase	*F. graminearum*
	0	CRV2T00019139_1	GH31	Glicoside hydrolase	
OG0000065	0	CRV2T00015284_1	GH3 + CBM1	Beta-glucosidase L-like protein	*H. solani*
OG0000078	0.02	CRV2T00012764_1		Uncharacterized	
OG0000079	0.006	CRV2T00011370_1		Uncharacterized	
OG0000080	0	CRV2T00011463_1	AA9 + CBM1 Predicted effector	Cellulose binding protein	
OG0000085	0.036	CRV2T00017150_1		Serine-type carboxypeptidase	
OG0000087	0	CRV2T00017010_1	GH39	Glicoside hydrolase	
	0	CRV2T00017011_1	GH39	Glicoside hydrolase	
	0	CRV2T00018900_1	GH39	Glicoside hydrolase	
	0	CRV2T00003298_1		Uncharacterized	
OG0000091	0.003	CRV2T00011446_1		Esterase	
OG0000092	0.007	CRV2T00014266_1	Predicted effector	Putative trypsin-like protease	*H. solani*
	0.007	CRV2T00017490_1	Predicted effector	Putative trypsin-like protease	
OG0000097	0.001	CRV2T00000045_1	GH28	Exopolygalacturonase	*H. solani*
OG0000098	0	CRV2T00008696_1		Glycosil hydrolase	
	0	CRV2T00012828_1		Glycosil hydrolase	
	0	CRV2T00013615_1		Glycosil hydrolase	
OG0000100	0.012	CRV2T00005946_1	GH95	Alpha-fucosidase	*H. solani*
OG0000105	0.005	CRV2T00011462_1	GH62 + CBM1 + CBM13	Arabinofuranosidase	*H. solani*
	0.005	CRV2T00018642_1	GH62 + CBM1 + CBM13	Aarabinofuranosidase	
OG0000107	0.005	CRV2T00002649_1		Aspartyl protease	
	0.005	CRV2T00009760_1		Aspartyl protease	
OG0000109	0.005	CRV2T00003747_1	GH35	Glycosil hydrolase	
OG0000112	0.008	CRV2T00014128_1		Esterase	
	0.008	CRV2T00018939_1		Esterase	
OG0000113	0.02	CRV2T00016251_1	GH28 Predicted effector	Endopolygalacturonase	*H. solani*
OG0000114	0.005	CRV2T00016193_1	Predicted effector	Uncharacterized	
	0.005	CRV2T00021563_1	Predicted effector	Uncharacterized	
	0.005	CRV2T00021564_1	Predicted effector	Uncharacterized	
OG0000118	0.018	CRV2T00018326_1	GH43 + CBM35	Glycosil hydrolase	*H. solani*
	0.018	CRV2T00021777_1	GH43 + CBM35	Glycosil hydrolase	
OG0000119	0.005	CRV2T00018325_1	GH31	Alpha/beta-glucosidase	
	0.005	CRV2T00021778_1	GH31	Putative alpha-glucosidase	
	0.005	CRV2T00021772_1	GH31	Putative alpha-glucosidase	
OG0000122	0.005	CRV2T00021100_1	GH3	Glycosil hydrolase	
	0.005	CRV2T00001147_1	GH3 + CBM1	Beta-glucosidase	*H. solani*
	0.005	CRV2T00021105_1	GH3 + CBM1	Beta-glucosidase	
OG0000125	0.018	CRV2T00013725_1	GH35	Glycosil hydrolase	
	0.018	CRV2T00012209_1	GH35	Putative beta-galactosidase	
OG0000127	0.005	CRV2T00010811_1		Metallocarboxypeptidase	
	0.005	CRV2T00020477_1		Metallocarboxypeptidase	
	0.005	CRV2T00020478_1		Metallocarboxypeptidase	
OG0000137	0	CRV2T00017782_1		Metallocarboxypeptidase	
	0	CRV2T00020443_1		Metallocarboxypeptidase	
	0	CRV2T00020444_1		Metallocarboxypeptidase	
OG0000193	0.007	CRV2T00010800_1	GH3 + CBM1	Glycosil hydrolase	
OG0000205	0	CRV2T00017226_1	GH79	Glycosil hydrolase	
OG0000217	0	CRV2T00004052_1		Cuticle-degrading protease	
	0	CRV2T00003815_1		Serine-type endopeptidase	

a Orthogroups were selected using the cut-off of one gene as minimum average gene count per species.

b Over expressed *C. rosea* in response to *F. graminearum* or *H. solani* based on previous results from Demissie et al. (2020) and Lysøe et al. (2017), respectively.

### 3.5. Identification and sequence analysis of CFEM proteins

Proteins with CFEM modules are considered to play an important role in fungi during interactions with other organisms (Srivastava et al., 2014; Zhang et al., 2015; Sabnam and Barman, 2017; Arya et al., 2020). The number of predicted CFEM proteins in *Clonostachys* varied from 21 in *Clonostachys* sp. CBS 192.96 to 32 in *C. chloroleuca*. However, the number of predicted secreted CFEM proteins ranged from only one in *Clonostachys* sp. CBS 192.96 to four in *C. chloroleuca* (Table 4). The total number of predicted CFEM proteins in *Clonostachys* spp. (21–32 genes) was higher compared with mycoparasitic *T. atroviride*, *T. virens* and *T. reesei* (14–17 genes). However, the CFEM gene copy number in the plant pathogenic *F. graminearum*, *F*. *verticillioides* and *F. vanetteni* (18–23 CFEM genes) was comparable to the number in *Clonostachys* spp. (Table 4). The predicted subcellular localization of CFEM proteins was found to be similar between *Clonostachys* and *Fusarium* species, with around half of them being transmembrane proteins, 40% GPI-anchored and only 8% secreted. In contrast, *Trichoderma* spp. CFEMs were predicted to have a different subcellular localization pattern, with 32% transmembrane proteins, 38% GPI-anchored and 17% secreted (Table 4). CFEM proteins may contain one or more copies of the CFEM module (Kulkarni et al., 2003). A conserved domain analysis identified a single CFEM module present in each predicted protein in the considered *Clonostachys* spp., except for one protein in each species that had two modules (Supplementary Table S4). Gene expression analysis identified seven genes coding for CFEM proteins in *C. rosea* that were upregulated in response to *F. graminearum* or *H. solani* (Supplementary Table S4).

**Table 4:** Number of proteins with CFEM domains found in the transmemebrane, GDP-anchored and secreted portion of the proteomes for each species of interest.

Species	Total CFEM	Transmembrane	GPI anchor	Secreted
*C. rosea*	22	11	8	3
*C. byssicola*	27	14	11	2
*C. chloroleuca*	32	18	10	4
*Clonostachys sp. 192.96*	21	10	10	1
*C. rhizophaga*	30	16	11	3
*C. solani*	21	10	10	1
*F. graminearum*	18	9	8	1
*F. vanetteni*	26	13	10	3
*F. verticillioides*	23	12	8	1
*T. atroviride*	17	6	4	3
*T. reesei*	14	4	7	3
*T. virens*	15	5	6	2
*N. crassa*	13	4	4	4

**Figure 5:**
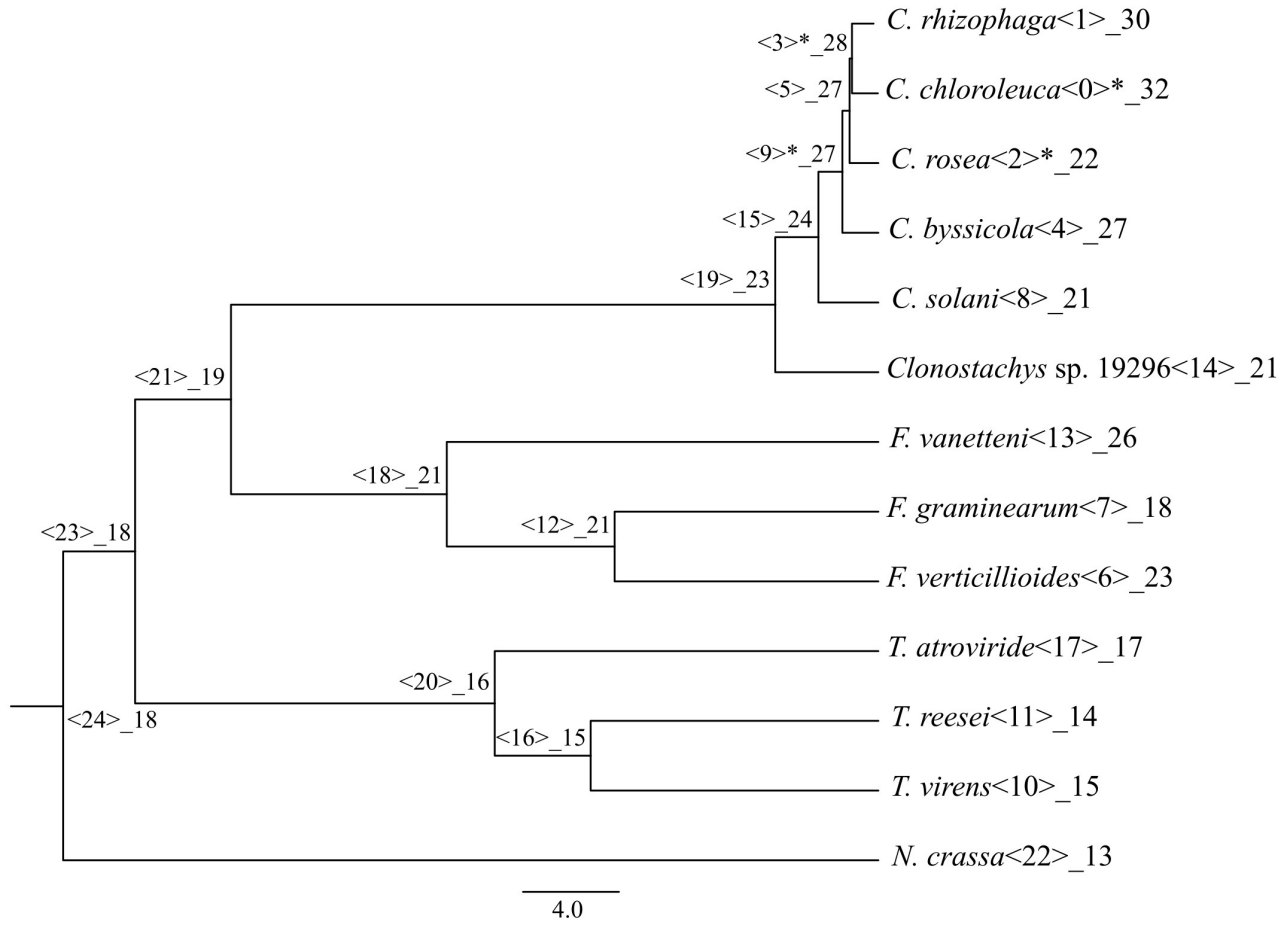
Number of CFEM proteins gained or lost during the evolution of the considered species, mapped on a phylogenetic tree obtained in Broberg et al. (2021). Significant changes are marked with the asterisk.

**Figure 6:**
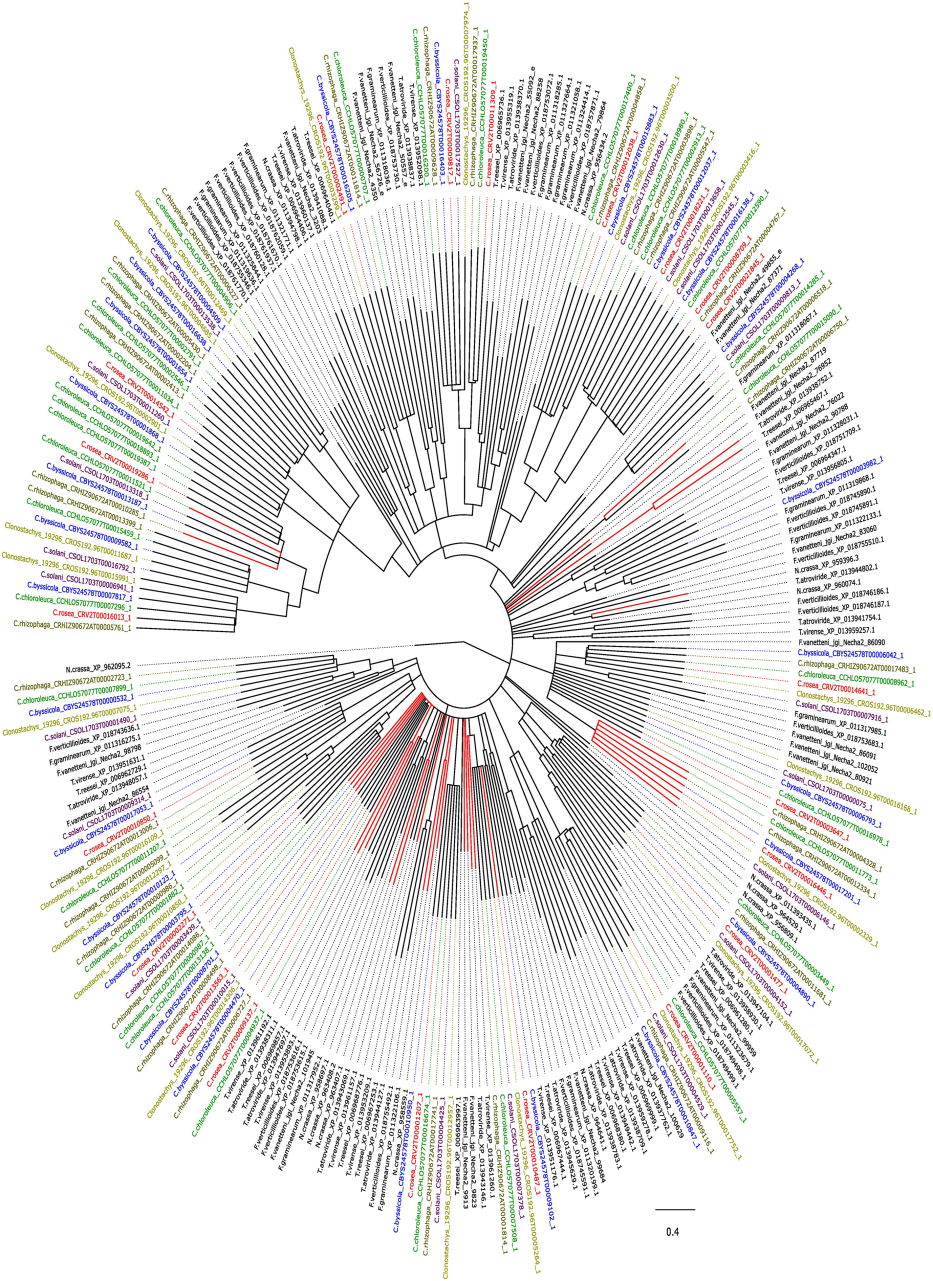
Phylogenetic tree showing the evolutionary relationship between the CFEM proteins in the species of interest. Red lines indicate secreted proteins. Bootstraps value lesser than 70% were condensed. The tree was generated with iqtree v.1.6.12 and visualized with figtree v.1.4.4.

An analysis with CAFE identified gene gains (*p* ≤ 0.05) in the ancestral lineage leading to *C. byssicola*, *C. chloroleuca*, *C. rhizophaga* and *C. rosea*, followed by additional gains in *C. chloroleuca* and losses in *C. rosea* (Figure 5). A phylogenetic analysis of predicted CFEM proteins from *Clonostachys* spp. together with above-mentioned species of *Trichoderma*, *Fusarium* and *N. crassa* showed that the CFEM proteins of *Clonostachys* spp. typically clustered in monophyletic groups, indicating recent diversification, even though some of them were orthologous to CFEM proteins in *Fusarium* and *Trichoderma* species (Figure 6). The phylogenetic tree further displayed low resolution among the deeper branches, sometimes in combination with incongruence with the species phylogeny, which may suggest a birth-and-death evolutionary process in combination with sequence divergence. Among the predicted CFEM proteins, the branches containing *C. rosea* proteins CRV2T00010850_1, CRV2T00012038_1, CRV2T00008709_1, CRV2T00021845_1, CRV2T00018221_1, CRV2T00019286_1, CRV2T00016013_1 and CRV2T00014542_1 were expanded in the *Clonostachys* genus (Figure 6), and they either had transmembrane domains or a GPI-anchor.

## 4. Discussion

The predicted secretomes of the considered *Clonostachys* spp. amount to 7.7% of their proteomes on average, more than what was predicted for *Trichoderma* spp. The secretomes comprise a similar number of predicted proteins in all the considered *Clonostachys* spp., with enriched GO terms relating to proteolysis, catabolism of carbohydrates and response to fungus. We therefore hypothesize a role of secreted proteins in nutrient acquisition and fungal antagonism, which correlates well with the fact that 18% of the *C. rosea* secreted proteins are encoded by genes that are differentially expressed during the response to plant pathogenic fungi (Lysøe et al., 2017; Demissie et al., 2018, 2020; Nygren et al., 2018). The proportionally higher number of proteases, lipases and oxidoreductases in *Clonostachys* spp., compared with *Trichoderma* spp., together with the proportionally lower number of CAZymes, suggests different evolutionary trajectories in the two genera, driven by differences in their ecological strategies. The *Clonostachys* secretome included many proteins involved in fungal cell wall catabolism, such as *C. rosea* chitinase ChiC2 (CRV2T00000260_1), whose gene deletion cause a reduction in the growth inhibitory activity of culture filtrates against *B. cinerea* and *Rhizoctonia solani* (Tzelepis et al., 2015). An ortholog of this gene is present in all *Clonostachys* spp. considered in this study. However, the total number of identified GH18 chitinases range between six and nine genes in *Clonostachys* spp., which is lower compared to the gene copy number (12–21 genes) predicted in *Trichoderma* spp. More specifically, the major difference between *Clonostachys* and *Trichoderma* relates to the number of subgroup C killer toxin-like chitinases, hypothesized to be involved in permeabilization of mycohost cell walls for toxin entry (Tzelepis and Karlsson, 2019), suggesting intrinsic differences in the mode of action of these mycoparasites. Another class of secreted CAZymes operating on fungal cell walls are GH16 endo-β-(1,3)-glucanases, with potential roles in cell wall morphogenesis and catabolism (Mouyna et al., 2016). This class is evolving for increased gene copy numbers in the analyzed *Clonostachys* spp. with on average nine genes, while only five are found in *Trichoderma* spp. It is possible that the abundance of GH16 enzymes in their secretomes allow *Clonostachys* spp. to better modify and adapt their cell wall for the interaction with their hosts, or simply indicate that GH16 endo-β-(1,3)-glucanases are involved in cell wall degradation of the fungal prey.

Likewise, several CAZyme families with a putative role in deconstruction of plant cell walls evolve for increased paralog numbers in *Clonostachys*, possibly mediating nutrient uptake or plant host colonization. Family AA9 is involved in the degradation of cellulose and the high number of AA9 genes has already been observed in both *C. rosea* and *C. byssicola* (Karlsson et al., 2015; Gomes et al., 2020). In the present study, we identified a large difference, between 16 and 32 predicted AA9 enzymes, in the secretomes of different *Clonostachys* spp. indicating an involvement in ecological niche adaptation. Moreover, AA9 was the most frequent CAZyme class in *C. rosea* secreted proteins involved in the response to plant pathogens. AA9 enzymes need exogenous electron donors to function correctly, and it has been hypothesized that these could come from AA3 glucose-methanol-choline oxidoreductases (Vaaje-Kolstad et al., 2010), which is also the most numerous class of secreted oxidoreductases in the considered *Clonostachys* species. Additional gene families evolving for gene gene gains or losses include GH31 and GH3 where many members are putatively involved in hemicellulose degradation, including glucosidases, xylosidases and alpha-L-arabinofuranosidases. Enzymes of this class are also present in CAZyme family GH43, which is the most abundant in *Clonostachys* spp. with up to 39 members, while another abundant class, GH28 (13 to 16 members), include enzymes predicted to degrade pectin (Markovič and Janeček, 2001). Cell-wall degradation results in oligomers, such as xyloglucan, which can serve as damage-associated molecular patterns (DAMPs) and activate plant immunity reactions, including pattern-triggered immunity (PTI) and induced systemic resistance (ISR), resulting in the defense-inducing activity of *Clonostachys* spp. (Beliën et al., 2006; Souza et al., 2017; Claverie et al., 2018). Interestingly, *Trichoderma* spp. had on average a lower number of GH28, GH3, GH31, GH43, AA9 and AA3 enzymes, again emphasizing the different mechanistic strategies that underlie the ecological opportunism of these two genera. It is possible that *Clonostachys* spp. perform their biocontrol action through a greater induction of defense on host plants through partial plant cell wall degradation, while *Trichoderma* spp. have a greater capacity for the direct degradation of the fungal cell wall. Additional studies are needed to confirm this. Recent proteomic studies have shown an increased production of secreted plant cell wall degradation enzymes in *Trichoderma* spp. upon contact with the plant hosts *Phaseolus vulgaris* and *Arabidopsis thaliana*, and *Clonostachys* spp. might behave similarly (González-López et al., 2021; da Silva et al., 2022). Alternatively, these genomic adaptations may indicate a greater capacity for saprophytic growth of *Clonostachys* spp., which may also influence its usefulness in biocontrol applications.

Another process facilitated by secreted glycoside hydrolases is plant root colonization. In particular, all the considered *Clonostachys* spp. have a homolog of the PG1 protein, a class GH28 CAZyme involved in tomato root colonization in *T. harzianum* (Morán-Diez et al., 2009), and the *C. rosea* homolog of this gene (CRV2T00004567_1) is involved in the response to *F. graminearum* (Demissie et al., 2020). Another gene family with members involved in the interaction with plant hosts is the hydrophobins, necessary for plant colonization and pathogenicity in the pathogen *M. oryzae* (Talbot et al., 1996; Kim et al., 2005). Among the secreted *C. rosea* hydrophobins, we identified *hyd3* (CRV2T00012494_1), a *F. graminearum* responsive gene (Demissie et al., 2020) whose deletion causes a reduction in root colonization (Dubey et al., 2014). This protein has a homolog in all considered *Clonostachys* spp., and it is similar (45% aa identity) to hydrophobin HFB2-6 of *T. asperelleum*, which has a function in root colonization and promotes jasmonic acid and salicylic acid signal transduction pathways in poplars (Huang et al., 2015). *C. chloroleuca* has almost twice as many hydrophobins, both secreted and otherwise, compared with other *Clonostachys* spp., suggesting that hydrophobins have evolved specific functions in the preferred ecological niche of this species.

Protease subfamily S8A is highly represented in the *Clonostachys* secretomes and it has previously been shown to evolve for gene gains in *C. rosea* (Iqbal et al., 2018a,b) and the same is true for the whole S8 family in *Trichoderma* spp. (Druzhinina et al., 2012). This family contains the serine endopeptidase subtilisin and its homologs, which have proven roles in biocontrol of fungi (Fan et al., 2014; Zhang et al., 2017) and nematodes (Ahman et al., 2002; Fekete et al., 2008). Numerous proteases of this class are proven to be involved in the response to mycohosts and nematodes, and this class was the most abundant one among the secreted *C. rosea* proteases found to be responsive to plant pathogens, together with class S1A. For example, the protease genes *prs6* and *prs16* are induced in *C. rosea* during the response to *F. graminearum* (Iqbal et al., 2018a,b; Demissie et al., 2020), while *prs11*, *prs14* and *prs16* are induced during parasitism of the potato pathogen *H. solani* (Lysøe et al., 2017). The serine protease *prC* gene is expressed in *C. rosea* when the fungus is degrading nematode cuticle material and is also involved in resistance to oxidative stress (Zou C. et al., 2010; Zou C.-G. et al., 2010). Many more members of these classes were detected in the secretomes of *Clonostachys* spp. than *Trichoderma* spp., indicating that *Clonostachys* spp. rely more on this type of proteases for their proteolytic action against mycohosts. However, serine proteases were also observed to be secreted in greater quantities upon *T. harzianum* interaction with *P. vulgaris*, suggesting a role in the interaction with the plant (da Silva et al., 2022).

The most numerous lipases in *Clonostachys* spp. proved to be GDSL-like lipases, which can potentially contribute to ethylene-based resistance in plants (Kwon et al., 2009; Gottwald et al., 2012). *C. rosea* and other species are known for inducing defense responses in plant hosts (Kamou et al., 2020; Sun et al., 2020), and this class of lipases could bolster that action. A subfamily of this class, GDSL esteraselipases exl3, are among the lipase families predicted to evolve for gene gains in *Clonostachys* species. Notably, we detected more than six *Clonostachys* GDSL-like lipases for each *Trichoderma* sp. in the secretomes, giving another indication that *Clonostachys* spp. have a greater part of their secretome dedicated to influencing plant hosts defense reactions. Among the most represented lipase families are also phospholipases A2, normally involved in nutrient acquisition but also in the modulation of host’s immune response (Köhler et al., 2006). One such gene (JK757061.1) is induced during *T. harzianum* colonization of tomatoes (Mehrabi-Koushki et al., 2012), and phospholipase A activity is a key mechanism by which *Trichoderma* spp. rupture the biological membranes of other fungi (Minchiotti et al., 2021).

*C. rosea* LysM protein LYSM2 (CRV2T00011102_1) is also predicted to be secreted. Deletion of the *lysm2* gene resulted in *C. rosea* mutants with impaired biocontrol capabilities towards *B. cinerea* and *F. graminearum*, and also altered the suppression of wheat defense genes *PR1* and *PR4* (Dubey et al., 2020). An ortholog of this gene is present in all considered *Clonostachys* spp. except for *C. rhizophaga*. Other secreted proteins of interest include homologs of the effector cerato-platanin protein EPL1, involved in induction of defense reaction in maize, cotton, beans and *Nicothiana bentamiana* (Djonović et al., 2006; Djonovic et al., 2007; Crutcher et al., 2015; Gomes et al., 2015; Cheng et al., 2018). The thioredoxin-like effector class is also present in the secretomes of all considered species, with three proteins present in all *Clonostachys* spp. and one (CRV2T00013356_1) involved in the response to *H. solani* in *C. rosea* (Lysøe et al., 2017). This class is normally involved in apoplastic reactive oxygen species scavenging to protect plant pathogens from oxidative stress during the interaction with the plant, and it could play a similar role in *Clonostachys* spp., which are known to withstand high amounts of oxidative stress (Viefhues et al., 2014; Li et al., 2016).

Among the orthogroups detected with Orthofinder, 38 are evolving for gene gains and 16 of them contain genes involved in the *C. rosea* response to either *F. graminearum* or *H. solani*. Among these, nine contain CAZymes putatively involved in hemicellulose degradation and seven include putative effectors. Effector proteins in biocontrol fungi are typically necessary to resist and suppress the defense responses of plant hosts in order to allow plant colonization (Mendoza-Mendoza et al., 2018; Nogueira-Lopez et al., 2018; Romero-Contreras et al., 2019). Additionally, these orthogroups include proteases involved in the degradation of the plant cuticle, which is fundamental to initiate defense responses (Xia et al., 2009; Aragón et al., 2017). Orthogroup OG0000113 in particular included homologs of TvPG2, an endopolygalacturonase regulating the induction of plant defense in *T. virens* (Sarrocco et al., 2017). Yet other orthogroups consisted of trypsin proteases, which are a part of the biological control action of *T. atroviride* (Grinyer et al., 2005)*. C. rosea* genes from these two orthogroups (CRV2T00016251_1 and CRV2T00014266_1) are both induced during the response to *H. solani* (Lysøe et al., 2017).

Several proteins with CFEM domains are predicted in the considered *Clonostachys* species. Such proteins are particularly numerous in fungal pathogens and they often act as cell-surface receptors, signal transducers, adhesion molecules or proteins involved in appressorium formation (Choi and Dean, 1997; Kulkarni et al., 2003; Zhang et al., 2015; Sabnam and Barman 2017). Interestingly, the considered *Clonostachys* spp. have higher numbers of CFEM proteins compared with *Trichoderma* species. In non-pathogenic fungi, CFEM can have a role in interactions with plants, and one member is upregulated in *T. atroviride* during plant host interaction (Guzmán-Guzmán et al., 2017), suggesting that their high number in *Clonostachys* spp. may be tied to a role in plant host perception and colonization. The high number of CFEM proteins could therefore help *Clonostachys* spp. to interact with a high variety of plant hosts, possibly by facilitating adhesion. Some of them, however, could also play a part in the interaction with mycohosts. In particular, the transmembrane CFEM protein CRV2T00016013_1 is part of a phylogenetic group evolving for gene gains in *Clonostachys* and is induced in *C. rosea* in response to *F. graminearum* (Demissie et al., 2020). Furthermore, the GPI-anchored protein CRV2T00009137_1 is the only CFEM protein encoded by a gene induced in response to both *H. solani* and *F. graminearum* (Lysøe et al., 2017; Demissie et al., 2020), indicating a general function in interspecific fungal interactions. This could be related to the known role of CFEM proteins as signal transducers (Kulkarni et al., 2003; Sabnam and Barman 2017).

## 5. Conclusion

This work investigates the composition of the predicted secretome of *Clonostachys* spp. and highlights its potential role in the mycoparasitic lifestyle and ecological opportunism of these commercially important fungi. Presence of proteins with a known role in fungal antagonism, including the chitinase CHIC2, the LysM protein LYSM2 and the endopolygalacturonase PG2 homolog, as well as several subtilisin-like peptidases and phospholipases A, emphasize the potential contribution of antibiosis to the biocontrol property of *Clonostachys*. *Clonostachys* spp. secretomes contained more CAZymes with a predicted function to degrade hemicellulose compared with *Trichoderma* spp., which however contained more chitinases. This may suggest that *Trichoderma* spp. are more adapted to degrade the cell wall of their mycohosts but less suited to induce defense reactions on their plant hosts. Moreover, we detected an unexpectedly high number of CFEM proteins in *Clonostachys* spp., typically more frequently found in plant pathogens, which also highlight the intimate interaction between *Clonostachys* spp. and plants, with potential consequences for their biocontrol activity. In summary, *Clonostachys* and *Trichoderma* species superficially share the same ecological lifestyle as rhizosphere-competent mycoparasites and opportunistic plant mutualists. Together with previous studies (Karlsson et al., 2015; Nygren et al., 2018; Broberg et al., 2021), the current work emphasizes several differences in the genomic characteristics of these two genera that show that convergent evolution resulted in adaptation of different underlying mechanisms for these apparent ecological similarities. This may have important consequences for the commercial exploitation of these fungi for biocontrol applications.

## Data availability statement

The original contributions presented in the study are included in the article/Supplementary material, further inquiries can be directed to the corresponding author.

## Author contributions

MD, MK, and DJ conceived and designed the analysis. EP and MG performed the analyses with inputs from MK and MD. EP, MG, DJ, MK, and MD discussed and interpreted the results. EP wrote the first draft of the manuscript. MD and MK revised the manuscript. All authors contributed to the article and approved the submitted version.

## Funding

This work was financially supported by the Swedish Research Council for Environment, Agricultural Sciences and Spatial Planning (FORMAS; grant numbers 2018-01420, 2021-01461), and Carl Tryggers Stiftelse för Vetenskaplig Forskning (CTS 19: 82).

## Conflict of interest

The authors declare that the research was conducted in the absence of any commercial or financial relationships that could be construed as a potential conflict of interest.

## Publisher’s note

All claims expressed in this article are solely those of the authors and do not necessarily represent those of their affiliated organizations, or those of the publisher, the editors and the reviewers. Any product that may be evaluated in this article, or claim that may be made by its manufacturer, is not guaranteed or endorsed by the publisher.
