# Supplementary figures and images for: Insights into the ecological generalist lifestyle of Clonostachys fungi through analysis of their predicted secretomes
Source: Front Microbiol. 2023 Feb 16;14:1112673. doi: 10.3389/fmicb.2023.1112673 (PMC9978495; doi:10.3389/fmicb.2023.1112673)

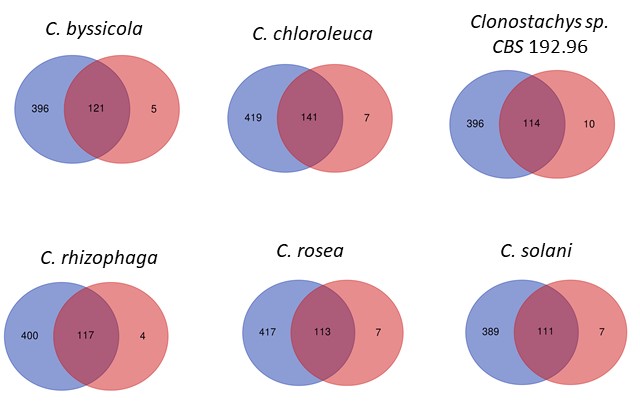

Supplement: Supplementary file 6 [file Image_1.JPEG]
